# Studies on the Manner of Collateral Regeneration From Nerve Stem to Motor Endplate

**DOI:** 10.3389/fphys.2021.795623

**Published:** 2022-02-28

**Authors:** Zhidan Qi, Dongdong Li, Li Li, Dexuan Meng, Jin Deng, Bo Jin, Xinyi Gu, Shen Wang, Chen Huang, Xiaofeng Yin

**Affiliations:** ^1^Department of Orthopedics and Trauma, Peking University People’s Hospital, Beijing, China; ^2^Department of Orthopedics, PLA Strategic Support Force Medical Center, Beijing, China; ^3^Peking University Third Hospital, Department of Orthopaedics, Beijing, China; ^4^Department of Intelligent Medicine, Pizhou People’s Hospital, Jiangsu, China

**Keywords:** peripheral nerve injury, collateral regeneration, neuromuscular junction, optical clearing, light-sheet microscopy, three-dimensional distributions

## Abstract

Despite recent evidence suggesting that nerve transfer techniques help improve clinical outcomes, the underlying manner by which collateral-regenerated nerve enters skeletal muscles to restore an organized pattern of the neuromuscular junction (NMJ) is unclear. To construct the animal models of collateral regeneration, the proximal peroneal nerve was fixed to the distal tibial nerve stump. Three months after surgery, the spatial distribution of motor endplates (MEPs) and corresponding in-muscle nerve branches in long flexor digitorum muscles were observed with tissue optical clearing combined with light-sheet microscopy in transgenic fluorescent mice. The results showed that the number of fibers in the proximal donor peroneal nerve was 415 ± 11, while regenerated nerve fibers in the distal tibial stump were 781 ± 43, which indicates a collateral regeneration ratio of 1.88. The spatial distribution of MEPs was restored to an organized pattern of the lamella, and the corresponding in-muscle nerve branches reverted to the normal manner such as after collateral regeneration. Beyond this, the numbers of MEPs dominated by the single distal nerve fiber were 25.58 ± 0.50 and 26.42 ± 0.94, respectively (*n* = 6, *p* > 0.05, collateral regeneration group vs. normal group). However, the numbers of distal-regenerated nerve fibers were less than those in normal control groups (781 ± 43 vs. 914 ± 55, *n* = 6, *p* < 0.05), and the number and perforations of MEPs were lower than those in normal control groups as such. In summary, this is the first study to show the manner of collateral regeneration of the peripheral nerve that the smaller proximal donor nerve can sprout more axonal buds to connect distal larger nerves and finally restore to an organized pattern of lamella dominated by corresponding in-muscle nerve branches.

## Introduction

Neuromuscular junctions (NMJs) are important structural and functional interfaces where skeletal muscle fibers receive neurotransmission signals from motor nerve terminals (MNTs) and trigger muscle contraction to perform physical activity (Hoke and Brushart, 2010; Pratt et al., 2013; Dorninger et al., 2017; Li et al., 2018). Peripheral nerve injury (PNI) can lead to the degeneration of nerve terminals (Osterloh et al., 2012; Gomez-Sanchez et al., 2017), and as the denervation time increases, the motor endplates (MEPs) become swollen and gradually degenerate, finally destroying the physiological structure of the NMJs and resulting in the poor functional recovery of skeletal muscles (Rich and Lichtman, 1989). Based on the potential for collateral regeneration of the peripheral nerve (Ide, 1996; Torigoe et al., 1996) and the development of microsurgical techniques and nerve tissue engineering (Haastert and Grothe, 2007), it provides a novel strategy to treat PNI by utilizing the compensatory potential of the smaller donor nerves (Shawe, 1955; Zhang et al., 2011; Gu et al., 2014; An et al., 2015; Yu et al., 2016; Zheng et al., 2018). As the structural basis of the muscular functional recovery following nerve repair, the underlying manner by which collateral-regenerated nerve enters skeletal muscles to restore an organized pattern of NMJ is unclear.

The clinical outcomes following nerve repair have long been measured by muscle strength or electrophysiology, but the further fine movement of the muscle also needs to be considered. Our recent study (Yin et al., 2019) showed that one skeletal muscle could be divided into one or several functional groups, mainly according to the distribution of the MEP lamella clusters, which are closely related to a more precise movement of the muscle. An unresolved question is whether collateral regeneration of the peripheral nerve changes such functional groups in the skeletal muscle. In addition, despite the collateral-regenerated nerves having a physiological function, the relationship between collateral-regenerated nerves and MEPs remains unclear, which is the fundamental objective for recovering the neurological function of skeletal muscles after nerve repair. Thus, a more comprehensive and precise approach is required to reveal the manner of collateral regeneration of the peripheral nerve.

In this study, we obtained three-dimensional (3D) images of the functional group consisting of all MEPs in one lamella cluster and the corresponding regenerated nerve branches by the optical clearing technique and ultramicroscopy to show the manner of collateral regeneration of the peripheral nerve.

## Materials and Methods

This study was carried out in accordance with the principles of the Basel Declaration and recommendations of *Chinese guidelines for the care and use of laboratory animals*. All experiments complied with the ARRIVE guidelines and were carried out in accordance with the National Institutes of Health Guide for the Care and Use of Laboratory Animals (NIH Publications No. 8023, revised 1978). The protocol was approved by the Ethics Committee of the Peking University People’s Hospital (Permit Number: 2020PHE089).

### Animal Models

**FIGURE 1:**
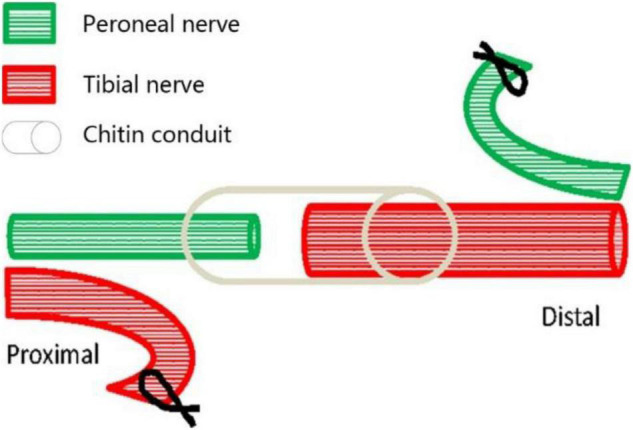
Surgical diagram of collateral peripheral nerve regeneration in group B. The proximal peroneal nerve is used to repair the distal tibial nerve. The peroneal nerve and tibial nerve are cut off, the proximal tibial nerve and distal peroneal nerve are sutured to nearby muscles, and then the distal tibial nerve is repaired by the proximal peroneal nerve through anastomosis with biodegradable chitin conduit.

Eighteen young female Thy1-YFP-16 mice weighing 20–30 g, obtained from the Jackson Laboratory (Maine, United States) and kept in the Laboratory Animal Centre of Peking University (Beijing, China), were used in this study. The mice were randomly divided into three groups, namely, the normal control group (A, *n* = 6), the proximal peroneal nerve-repaired distal tibial nerve group (B, *n* = 6), and the unrepaired tibial nerve group (C, *n* = 6). The animals were anesthetized by 1.5% isoflurane inhalation. The surgical procedures were performed on the right peroneal nerves and the tibial nerves, using standard microsurgical techniques under aseptic conditions. First, the peroneal nerve, the tibial nerve, and the sural nerve were exposed and freed from the surrounding tissue by gentle dissection. The right peroneal and tibial nerves were transected about 2 mm distal to their origins from the sciatic nerve. Second, in group B (Figure 1), the proximal tibial nerves and distal peroneal nerves were ligated and sutured to nearby muscles with 7-0 nylon sutures. Third, the proximal peroneal nerve was used to repair the distal tibial nerve. The free proximal and distal stumps of the repaired nerve were inserted (1 mm) into a biodegradable chitin conduit (tube length: 3 mm, thickness: 0.1 mm, inner diameter: 0.6 mm) and fixed with 11-0 nylon sutures. The gap between the repaired nerve stumps was 1 mm. Biodegradable chitin conduits, made of a polysaccharide shell, are biocompatible and biodegradable artificial nerve grafts (Jianping et al., 2012). In group C, the peroneal and tibial nerves were transected and left unrepaired. At the end of the operation, the surgical site was closed in layers with 5-0 nylon sutures. Group A just exposed the nerve but did not transect the nerve. The animals were maintained on standard mice chow and water, which were available *ad libitum*, under a 12-h light-dark cycle and standardized housing conditions before and after the operation.

### Histological Assessment for Nerve Regeneration

The distal tibial nerves and long flexor digitorum (LFD) branches of the tibial nerves were obtained at 3 months after the surgery, to assess nerve regeneration. In addition, the proximal peroneal nerves were acquired to calculate the collateral regeneration ratio in group B. These nerves were postfixed in 4% paraformaldehyde at 4°C overnight and then dehydrated in 10, 20, and 30% sucrose for 12 h. Consecutive frozen transverse sections measuring 5 μm were cut using a Cryostat Microtome (Leica CM 1950, Germany). The nerve sections were mounted onto microscope slides with glass coverslips. The sections were imaged under a fluorescence microscope (Leica DM 4B, Germany) with a 20 × microscope objective with an excitation wavelength of 488 nm. The number of fluorescent nerve fibers was determined by a manual count.

### Labeling of Motor Endplates and Tissue Optical Clearing

**FIGURE 2:**
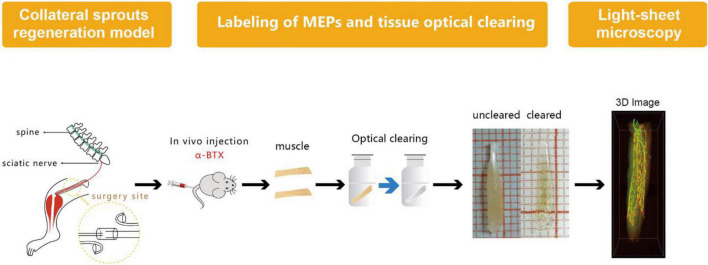
Diagram of labeling of MEP, tissue optical clearing, and light-sheet microscopy. MEPs were labeled using α-bungarotoxin at 3 months after the surgery. Then, the long flexor digitorum was subjected to the optical clearing to obtain three-dimensional (3D) images of MNTs and MEPs using light-sheet microscopy.

Labeling of MEPs was performed to display the structure of regenerated NMJs, using Alexa Fluor 647 α-bungarotoxin (Invitrogen, New York, United States), which was injected *via* the caudal vein (Chen et al., 2016). First, 1 h after injecting the α-bungarotoxin (0.3 μg/g), the mice were perfused transcardially with a normal saline solution followed by a 4% paraformaldehyde solution. Second, the intact LFD was dissected and postfixed with 4% paraformaldehyde at 4°C overnight. Third, the dissected and postfixed muscle was subjected to optical clearing employing the 3DISCO technique (Erturk et al., 2012; Richardson and Lichtman, 2015). During the clearing procedure, the fixed muscle was subjected to dehydration in 50, 70, 80, and 100% tetrahydrofuran and then treated with 100% dibenzyl ether to make it completely transparent (Figure 2). The sample was placed on a shaker for 2.5 h, with each clearing step set at 4°C.

### Confocal Microscopy

After optical clearing, the transparent muscle was placed on a cover glass and imaged under an inverted confocal microscope (ZeissLSM710, Oberkochen, Germany). The fluorescent signals of the MEPs and MNTs were captured with excitation wavelengths of 647 and 488 nm, respectively. Three microscopic fields were randomly chosen in each muscle to acquire 40 μm thick stacks in the *Z*-axis (1 μm steps) with a × 10 microscope objective. The 2D images of the MNTs and MEPs were acquired by maximum-intensity projection (MIP, thickness = 40 μm) reconstruction using the ImageJ software (NIH, Bethesda, MD), and then the two MIP images were merged to yield a composite image that displayed the structure of NMJs. Only NMJs with clearly visible MNTs and MEPs were scored. The MIP images were scored as follows: 10 NMJs were scored per mouse for perforation and occupancy counts and 20 for NMJ area measurements. A perforation is defined as a region where there is no observable α-bungarotoxin staining within MEPs. NMJ occupancy was calculated as a percentage of MNTs area to MEPs area × 100%.

### Light-Sheet Microscopy

**FIGURE 3:**
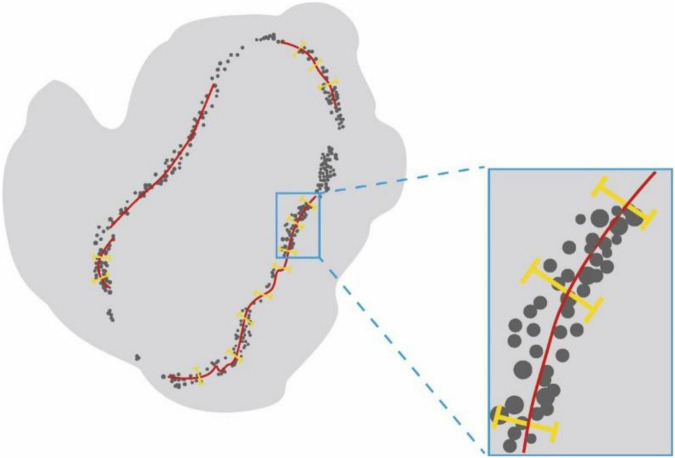
The schematic diagram for measurement of the width of NMJs lamella clusters. Schematic diagram showing the cross-sectional images of LFD. Lamella clusters of MEPs in cross-sections were indicated by gray dots. The red line identifies the MIP images of nerve fibers in the cross-section. The yellow line represents the width of lamella clusters.

After confocal microscopy, the intact muscles were imaged *via* light-sheet microscopy by an ultramicroscope equipped with an MV PLAPO 2X/0.5 dry objective (working distance: 20 mm). This technique was used to obtain 3D images of MNTs and MEPs in intact LFD. The *Z*-axis step size was 5 μm (Figure 2). The acquired images were analyzed using the Imaris software (Bitplane, Switzerland), whereby the 3D distributions of NMJs were reconstructed, and the numbers of NMJs were counted. The 3D visualization and quantification were performed *via* the spots and surfaces using the Imaris software. To measure the width of the lamella clusters, we also obtained the cross-sectional images of LFD. Then, the MIP of Z-stacks (thickness = 250 μm) was developed using the ImageJ software. For each MIP image, five positions were measured, and the maximum value of the MIP image was used to calculate the bandwidth of the NMJs (Figure 3).

### Statistical Analysis

The SPSS 20.0 software (SPSS Inc., United States) was used for the statistical analysis. Independent *t*-tests and one-way ANOVA followed by Student-Newman-Keuls tests were adopted for two-group and multigroup comparisons, respectively. All data are expressed as the mean ± SD; differences were considered statistically significant at *p* < 0.05.

## Results

### The General Condition of the Mice

All the mice survived in this study. None of the mice showed toe self-biting or ulcers in the operated limbs. Mice in groups B and C had lameness, movement disorders, and varying degrees of muscle atrophy in the right limbs. The wet weight of the LFD in groups A, B, and C was 32.7 ± 2.6, 21.5 ± 2.7, and 10.9 ± 0.9 mg, respectively. (A: normal control group, B: proximal peroneal nerve-repaired distal tibial nerve group, and C: unrepaired tibial nerve group, *n* = 6 for each group, *p* < 0.05 for both).

### Histological Assessment for Nerve Regeneration

Quantification of the nerve fiber numbers demonstrated that the numbers of distal tibial nerve fibers and LFD branches of the tibial nerve fibers were less in the nerve-repaired group than in the normal control group (Table 1) (914 ± 55, 220 ± 6, in the normal control group; 781 ± 43, 180 ± 11, in the nerve-repaired group, *p* < 0.05). The number of proximal peroneal nerve fibers was significantly less than distal tibial nerve fibers in the nerve-repaired group (415 ± 11 vs. 781 ± 43, *p* < 0.05). No fluorescent signals were detected in the distal nerves in the unrepaired tibial nerve group (Table 1).

**TABLE 1:** Comparisons of the number of nerve fibers and NMJs across all groups.

Group	MEPs number	FDL numbers	R_NF_	Proximal axon numbers	Distal axon numbers	Collateral regeneration ratio	Relative number of a nerve fiber innervating MEPs
Group A	5,624 ± 240	220 ± 6	25.58 ± 0.50	914 ± 55	914 ± 55	1.00	25.5 ± 0.50
Group B	4,765 ± 391*	180 ± 11*	26.42 ± 0.94	415 ± 11*	781 ± 43*	1.88	49.67 ± 1.46*
Group C	781 ± 43	NA	NA	NA	NA	NA	NA

The number of proximal tibial nerve fibers was considered to be equal to the number of distal tibial nerve fibers in group A. MEP, motor endplate; FDL, LFD branch of tibial nerve fibers; R_NF_, ratio of the number of MEPs to the number of FDLs; collateral regeneration ratio (CRR) = ratio of the number of distal axons to the number of proximal axons; relative number of a nerve fiber innervating MEPs = RNF × CRR. *p < 0.05 group B vs. group A (A, normal control group; B, proximal peroneal nerve-repaired distal tibial nerve group; and C, unrepaired tibial nerve group, n = 6 for each group).

### Rehabilitation of Spatial Distribution of Neuromuscular Junctions

**FIGURE 4:**
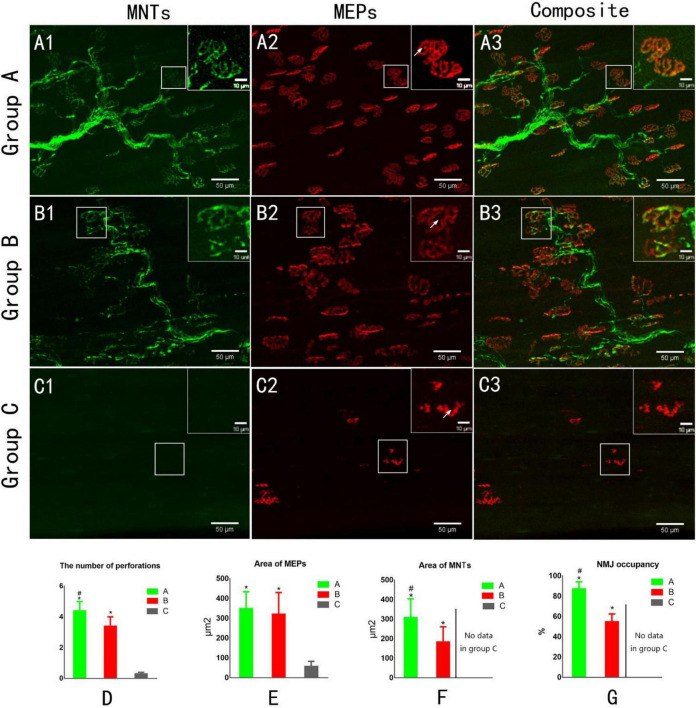
Confocal microscopy to assess reinnervation of NMJs **(A1,B1,C1)**. The yellow fluorescent protein is visualized in motor axons to demonstrate motor nerve terminals (MNTs) **(A2,B2,C2)**. Motor endplates (MEPs) are stained with a-bungarotoxin **(A3,B3,C3)**. Composite images of MNTs and MEPs. MEPs appear “pretzel-shaped” both in groups A and B. The MEPs are degenerated, appearing “plaque-shaped,” and no MNT signals are detected in Group C **(C1–C3)**. White arrows indicate the perforation of MEP **(A2,B2,C2)**. **(D)** The number of perforations per MEP. **(E)** The area of MEPs. **(F)** The area of MNTs. **(G)** NMJ occupancy (MNT area/MEP area × 100%). *p < 0.05 vs. C group, ^#^*p* < 0.05 vs. B group. (A, normal control group; B, proximal peroneal nerve-repaired distal tibial nerve group; and C, unrepaired tibial nerve group, *n* = 6 for each group, bars = SD).

**FIGURE 5:**
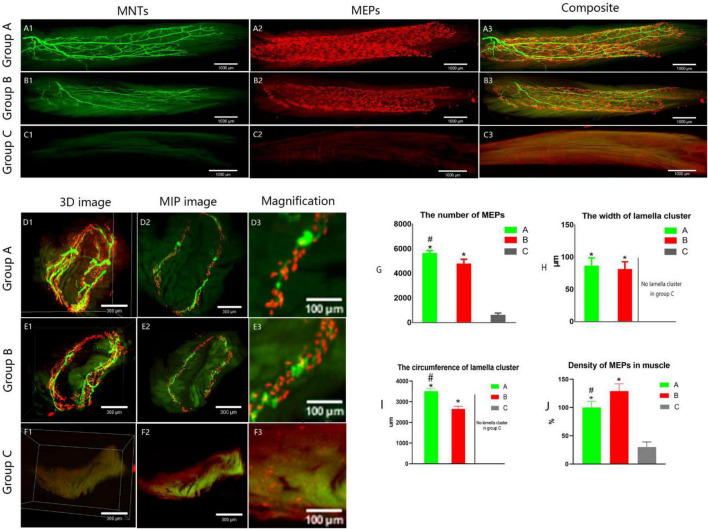
Spatial distribution of the neuromuscular junctions in LFD. AChRs aggregates in the post-synaptic membrane were stained with 647-α-bungarotoxin (α-BTX), a very potent ligand for AChR (visualized in red). The terminal axon of motor neuron was labeled using Thy1-YFP-16 mice (shown in green). **(A1–C3)** The three-dimension distribution of NMJs. Calibration bar: 1000 μm. **(D1–F3)** The cross-sectional images of NMJs. Panels **(D3, E3, F3)** show the width of the MEP lamellas which were the enlarged images (**D2, E2, F2**, respectively). Calibration bar: 300 and 100 μm. **(G)** The total number of MEPs in LFD, **(H,I)** The width and the circumference of lamella cluster of NMJs. There is no obvious lamella cluster in C group **(F1–F3)**. **(J)** The density of MEPs in LFD (ratio of the total number of MEPs to the volume of FDL). (A, normal control group, B, proximal peroneal nerve-repaired distal tibial nerve group, and C, unrepaired tibial nerve group, *n* = 6 for each group, *p < 0.05 vs. C group, ^#^*p* < 0.05 vs. B group. Bars = SD).

It showed that collateral regenerated axons could regain their distribution in lamella clusters (group B, Figures 4B1–B3 and Supplementary Video 1) as normal animals (group A, Figures 5A1–A3), while there was an irregular spatial distribution of NMJs in unrepaired group (group C, Figures 5C1–C3). The lamella width and circumference were 86 ± 13, 3508 ± 130 and 81 ± 12, 2648 ± 133 μm (Figures 5H–I) in normal animal group and collateral regeneration group, respectively (*n* = 6 for each group, Figures 5D1–D3, and Figures E1–E3). There were no obvious MEP lamella clusters in the C group (Figures 5F1–F3). However, there was a significant difference in the number of MEPs among groups A, B, and C (group A, 5,624 ± 240; group B, 4765 ± 391; group C, 600 ± 187; *n* = 6 for each group, *P* < 0.05) (Figure 5G). Interestingly, due to muscle atrophy, the density of MEPs in muscle was instead increased (Figure 5J).

### Regeneration of Neuromuscular Junctions

Confocal microscopic images revealed the structure of MNTs (shown in green) and MEPs (shown in red). The images of MEPs appeared “pretzel-shaped” in both the control group and nerve-repaired group, while the MEPs were irregularly shaped in the unrepaired group (Figures 4A2,B2,C2). The collateral-regenerated axons restored the structure of NMJs (Figures 4B1–B3), though the fluorescent signal was less heterogeneous compared with that of the control group (Figures 4A1–A3). The maturation of NMJs was assessed by counting the number of perforations of a single MEP. It was 4.4 ± 0.6 and 3.4 ± 0.6 in the control group and nerve-repaired group, respectively (*n* = 6 for each group, *p* < 0.05, Figure 4D).

The areas of MNT (Figure 4E) and MEP (Figure 4F) were 309 ± 95/350 ± 84 and 184 ± 77/321 ± 109 μm^2^ in the control group and nerve-repaired group, respectively. The MEP area showed no difference in both the control group and the nerve-repaired group. The MNT area in the nerve-repaired group was smaller than that in the control group (*n* = 6 for each group, *p* < 0.05). We also observed a significant difference in the NMJ occupancy (the ratio of MNTs to MEPs area), which was 87.3 ± 6.8 and 55.0 ± 7.5% in healthy muscle and the collateral regeneration muscles (*n* = 6 for each group, *p* < 0.05) (Figure 4G). The normal structure of MEPs was not observed in the unrepaired group (Figures 4C1–C3).

## Discussion

As an important therapy to help improve clinical outcomes, the nerve transfer technique essentially involves repair of the distal denervated nerve element by an adjacent foreign donor nerve. Previous studies focused more on functional recovery by muscle strength or electrophysiology, however, the finer 3D structure of regenerated nerve branches and MEPs following this nerve repair remains unclear.

Collateral regeneration of peripheral nerve restores an organized pattern of lamella clusters of MEPs. The novel and convenient labeling technique for MEPs with optical clearing and light-sheet microscopy were utilized in this study to find the distribution of NMJs in un-sectioned LFD. This labeling and imaging method avoids the common problems resulted from traditional histological methods (Aquilonius et al., 1984; Prodanov et al., 2005), which are laborious and time-consuming and may result in loss of structural information. In particular, the novel technique could show a “lamellar” distribution of MEPs in the skeletal muscle, which was related with a more precise movement of the muscle in our previous study (Shawe, 1955). In this study, spatial distribution of MEPs and lamella width were no significant difference between repair group and control group (Figure 2). It indicated motion pattern of renervated muscle unchanged. In our opinion, the assessment of muscular functional recovery is not only seen in general muscle strength in prevous studies, but also in recovery of fine-scale adjustments of the muscular motion. In addition, we must also be aware that although the motor neurons in the spinal cord after nerve repair were switched from tibial motor neuron to peroneal motor neuron, the distribution of terminal axons and MEPs remained the same co-locatation. It indicated that it mainly was the regenerated terminal axons that controlled the recovery of distribution of MEPs on muscle fibers. The spinal motor neurons might only provide necessary electrical stimulation or chemical stimulation to prevent synapse elimination.

**FIGURE 6:**
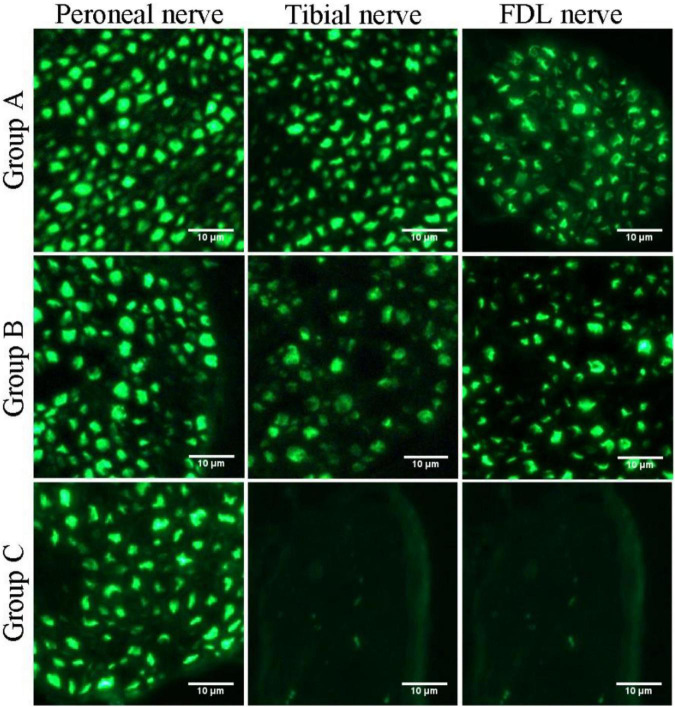
Fluorescence microscopy images of transverse sections of the peroneal nerve, tibial nerve, and long flexor digitorum branch of the tibial nerve (FDL nerve).

**FIGURE 7:**
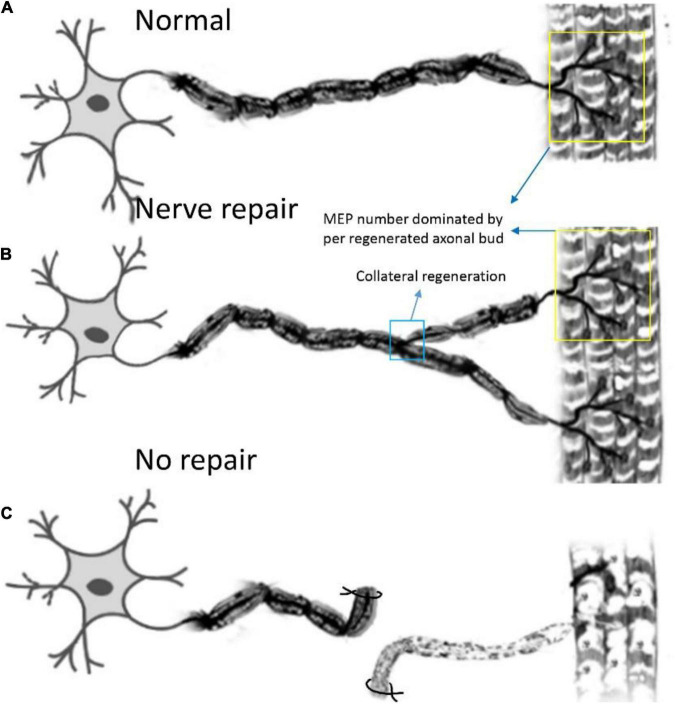
Pattern diagram displaying the reinnervation pattern. **(A)** The normal control pattern of the motoneuron. **(B)** One motoneuron sprouts about two axon buds to reinnervate the motor endplates, and the control region of one motoneuron is augmented after collateral regeneration, while the mean number of MEPs, dominated by regenerated axonal buds, remains unchanged. **(C)** The muscle is denervated due to axonal injury.

In this study, we first elucidated a complete manner of the collateral regeneration from nerve stump to motor axon terminals. To begin with, at the nerve stump, the number of nerve fibers in the donor nerve was 415 ± 11, while that in the recipient nerve was 781 ± 43. The fluorescence microscopy images of nerve stump was shown in Figure 6. That is to say, one donor nerve sprouts an average of 1.88 axonal buds, whereas a neuron in physiological state only sprouts one axon. Secondly, the manner of collateral regenerated terminal axons in skeletal muscle was similar as normal (Figures 3A1–B1). The regenerated terminal axons restored dendritic spatial distribution by lateral extensions from the suture site. However, the fluorescence intensity becomed weaker and the regenerated terminal axons becomed more sparse, which corresponded to the less MEPs. In addition, the number of MEPs dominated by regenerated single distal nerve fiber was approximately the same at the neuromuscular junction (25.58 ± 0.50 in collateral regeneration group vs. 26.42 ± 0.94 in normal group). Therefore, the compensatory amplification did not occur at neuromuscular junction and the collateral regenerated motor axon terminals innervated the same MEPs. The reinnervation pattern of peripheral nerve by collateral regeneration was revealed in Figure 7.

The number of perforations per MEP and NMT occupancy at neuromuscular junction were not fully restored. Generally speaking, the MEPs become unstable (t_1/2_ ≈ 1 day) in denervated muscles after nerve injury (Sleigh et al., 2014b) until regenerating axons reinnervate the MEPs after *in-situ* repair (Sleigh et al., 2014a). And the denervated MEPs transitioned from a relatively simple, plaque-like shape to a complex, pretzel-like structure, allowing efficient acetylcholine release and receptor binding. As NMJs age, AChR clusters break into fragments that could be poorly innervated and many disorders affecting motoneurons, muscles, and SCs are associated with NMJ decline. Based on the switch of MEPs, we also assessed the maturation of the MEPs by counting the number of perforations per MEP and MNT occupancy, which were high in healthy muscle (Marques et al., 2000; Chao et al., 2013). Our study found that the number of perforations per MEP and NMT occupancy were increased after collateral regeneration, but less than unrepaired group, indicating that collateral regeneration could restore the structure of single MEP and promoted the maturation of MEPs, despite not restoring to normal levels. This is perhaps because individual motor neuron can only innervate a limited number of MEPs and NMTs. In particular, peroneal motor neurons have to replace tibial motor neuron to innervate more MEPs and NMTs, which might be out of the maximum ability range and result in limited structural recovery. Of course, the short recovery time maybe perhaps one of the reasons.

Due to these structural changes of collateral regeneration above, the neuromuscular transmission failure (NMTF) should also be taken into consideration, which was used as the measure of muscular function. In a study, we used different axon number of the proximal tibial nerve to serve as the donor nerve and then fixed together with the distal stump. To assess the physiological function of these regenerative collaterals, we performed the electrophysiological experiment and found that the waveform had 1 peak before reconstructive surgery, while most had more than 1 peak following surgery (Salpeter and Loring, 1985). These results suggest that there is heterogeneity of the electrophysiological properties among the regenerative axons. In another study, we explored the relationship between different innervation ratio and skeletal muscle function, such as maximal compound muscle action potential (CMAP) amplitude and complete tetanic tension (Feng et al., 2000). We found that within a certain level of nerve injury, the skeletal muscle function maintained basically unchanged via complete compensation of motor endplates, but when the nerve injury level was over approximately 85%, the skeletal muscle function was totally lost.

When interpreting our findings, several limitations should be considered. In this study, Thy1-YFP-16 mice expressing yellow fluorescent protein in all motor and sensory neurons (Brushart, 1988) were used to conveniently and accurately count total fluorescent nerve fiber numbers and show the structure of NMJs and their spatial distribution. However, we counted all fluorescent nerve fibers, without distinguishing motor neurons from sensory neurons. Further, our study investigated the effects of collateral regeneration only at one time point. As the reinnervation time increases, some regenerated nerve fibers may gradually diminish due to their inefficient connections with effectors (Rich and Lichtman, 1989; Brushart, 1993; Jianping et al., 2012). In addition, residual denervated MEPs may exist in the LFD, which may affect the number and spatial distribution of NMJs. Therefore, further studies may be required to explore the long-term effects of collateral regeneration on NMJs. Besides, we elucidated the manner of non-collateral regeneration in a recent study and found that the spatial distribution of NMJ was similar to normal group (Chen et al., 2016). And about the structure of regenerating MEPs, there was no differences between the immediate repair and control groups in the shape and the mean area of MEPs.

Some interesting factors which may influence collateral regeneration need to be further explored, such as gender, species and different fiber types. Previous study showed that the gender likely had an effect on the nerve regeneration (Erturk et al., 2012). In addition, in our previous studies, we tried different types of nerve repairs in different animal models. For example, in the rhesus monkey model, we established distal ulnar nerve defects and repaired using muscular branches of the right forearm pronator teres. The rate of multiple amplification of regenerating myelinated nerve fibers was 1.6132. In addition, in a rabbit model, proximal median nerve was served as the donor nerve to repair the distal median and whole ulnar nerve. The ratio of proximal donor axon number to the distal acceptor nerve axon number is about 1:1.6933. In the collateral-regenerated models of rats, the ratio of distal regenerative myelinated axon number to proximal donor nerve axon number was 1.8334. According to the studies above, we have the following conclusion: (1) the number that the diatal regenerative axons can be greater than that of the proximal donor axons if providing the necessary condition; (2) the neuron of upper or lower extremities can both regenerate and maintain more than one collateral in a regenerative distal stump in different species; and (3) this amplifying ratio increased as the RDP increased and had a theoretical maximum value of approximately (Hoke and Brushart, 2010; Dorninger et al., 2017). It will be interesting to explore whether there is difference in the manner of collateral regeneration between different species and different fiber types.

## Conclusion

Our study shows the manner of collateral regeneration of peripheral nerve that the smaller proximal donor nerve can sprout more axonal buds to connect distal larger nerves at the axonal level and finally restored to an organized pattern of lamella dominated by corresponding in-muscle nerve branches. The study also elucidates that collateral regeneration did not change the inherent spatial pattern of neuromuscular junctions despite alteration of motor neurons.

## Data Availability Statement

The original contributions presented in the study are included in the article/Supplementary Material, further inquiries can be directed to the corresponding author/s.

## Ethics Statement

The animal study was reviewed and approved by the Ethics Committee of the Peking University People’s Hospital (Permit Number: 2020PHE089).

## Author Contributions

ZQ and LL performed most of the experiments with the help of DM and JD. BJ and XG performed statistical analysis. SW and CH wrote the manuscript.XY initiated, conceived, and supervised the project, with the help of DL. All authors contributed to the experimental design and interpretation and commented on the manuscript.

## Conflict of Interest

The authors declare that the research was conducted in the absence of any commercial or financial relationships that could be construed as a potential conflict of interest.

## Publisher’s Note

All claims expressed in this article are solely those of the authors and do not necessarily represent those of their affiliated organizations, or those of the publisher, the editors and the reviewers. Any product that may be evaluated in this article, or claim that may be made by its manufacturer, is not guaranteed or endorsed by the publisher.
