# Supplementary material for: Studies on the Manner of Collateral Regeneration From Nerve Stem to Motor Endplate
Source: Front Physiol. 2022 Feb 28;12:795623. doi: 10.3389/fphys.2021.795623 (PMC8919963; doi:10.3389/fphys.2021.795623)
Supplement: Supplementary file 1 [file Table_1.DOCX]

**Effects of collateral regeneration of peripheral nerve on spatial distribution of neuromuscular junctions in mice**

Dongdong Li^1,3#^, Xuefeng Zhou^2#^, Shuai Han^1^, Bo Jin^1^, Bo Chen^1^, Yusong Yuan^1^, Xinyi Gu^1^, Baoguo Jiang^1^, Xiaofeng Yin^1^*

^1^Department of Orthopedics and Trauma, Peking University People’s Hospital, Beijing, China

^2^Department of Orthopedics, PLA Strategic Support Force Medical Center, Beijing, China

^3^ Department of Surgery, the 517^th^ Hospital of the People’s Liberation Army, Xinzhou, Shanxi, China

**^#^**Equal contributors and co-first authors.

*Correspondence: Xiaofeng Yin, [xiaofengyin@bjmu.edu.cn](mailto:xiaofengyin@bjmu.edu.cn)

**Supplementary video.** 3D reconstruction of NMJs in Long Flexor Digitorum.
